# Recent Advances in the Treatment of Insulin Resistance Targeting Molecular and Metabolic Pathways: Fighting a Losing Battle?

**DOI:** 10.3390/medicina58040472

**Published:** 2022-03-25

**Authors:** Marta Wolosowicz, Slawomir Prokopiuk, Tomasz W. Kaminski

**Affiliations:** 1 Department of Physiology, Medical University of Bialystok, 15-222 Bialystok, Poland; marta.wolosowicz@umb.edu.pl; 2 Faculty of Health Sciences, Lomza State University of Applied Sciences, 18-400 Lomza, Poland; sprokopiuk@ansl.edu.pl; 3 Department of Medicine, Pittsburgh Heart, Lung and Blood Vascular Medicine Institute, University of Pittsburgh, Pittsburgh, PA 15260, USA

**Keywords:** insulin resistance, type 2 diabetes, glucose metabolism, obesity, metabolic syndrome

## Abstract

Diabetes Mellitus (DM) is amongst the most notable causes of years of life lost worldwide and its prevalence increases perpetually. The disease is characterized as multisystemic dysfunctions attributed to hyperglycemia resulting directly from insulin resistance (IR), inadequate insulin secretion, or enormous glucagon secretion. Insulin is a highly anabolic peptide hormone that regulates blood glucose levels by hastening cellular glucose uptake as well as controlling carbohydrate, protein, and lipid metabolism. In the course of Type 2 Diabetes Mellitus (T2DM), which accounts for nearly 90% of all cases of diabetes, the insulin response is inadequate, and this condition is defined as Insulin Resistance. IR sequela include, but are not limited to, hyperglycemia, cardiovascular system impairment, chronic inflammation, disbalance in oxidative stress status, and metabolic syndrome occurrence. Despite the substantial progress in understanding the molecular and metabolic pathways accounting for injurious effects of IR towards multiple body organs, IR still is recognized as a ferocious enigma. The number of widely available therapeutic approaches is growing, however, the demand for precise, safe, and effective therapy is also increasing. A literature search was carried out using the MEDLINE/PubMed, Google Scholar, SCOPUS and Clinical Trials Registry databases with a combination of keywords and MeSH terms, and papers published from February 2021 to March 2022 were selected as recently published papers. This review paper aims to provide critical, concise, but comprehensive insights into the advances in the treatment of IR that were achieved in the last months.

## 1. Introduction

**Figure 1:**
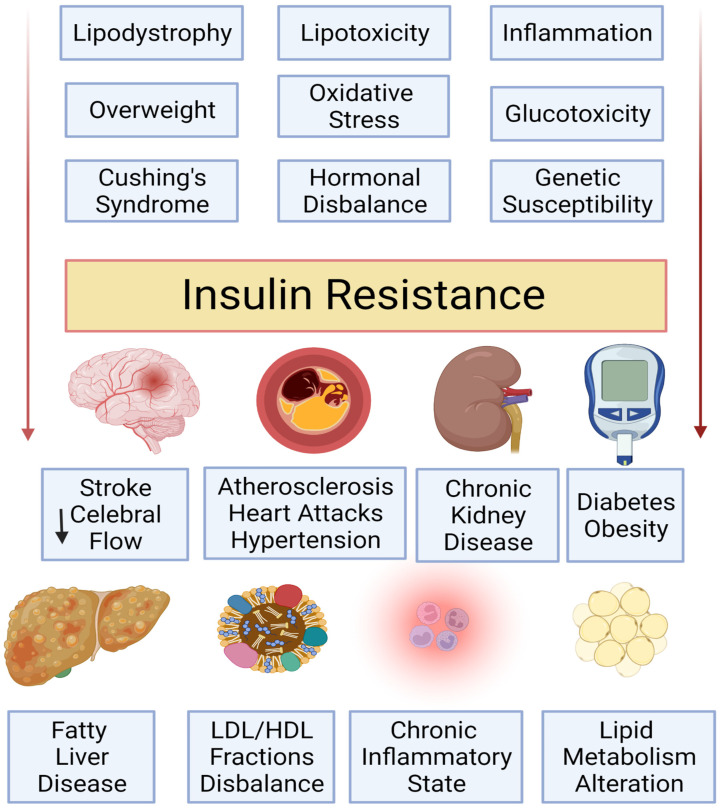
Overview of insights into the most common causes and main outcomes of IR. Insulin resistance is caused by a plethora of metabolic changes in lipid, glucose and hormone status as well as chronic oxidative stress and a chronic inflammatory state [2,3]. The main outcomes include, but are not limited to, increased risk of CVD, alterations in lipid and glucose metabolism, and lower life quality in general [4,5].

Diabetes is a chronic disorder that was the first non-communicable disease to be recognized by the United Nations as a 21st-century pandemic, and it develops either when the pancreatic β-cells do not secrete enough insulin or when the cells cannot productively use circulating insulin. Insulin is a hormone that maintains normal blood glucose levels by facilitating cellular glucose uptake and regulating carbohydrate, lipid, and protein metabolism. Hyperglycemia, commonly known as raised blood sugar level, is a common outcome of developing diabetes mellitus and continuously leads to mild-to-severe damage to many body organs, especially the nerves and blood vessel structures [1,2,3]. Insulin resistance (IR) is described as a defective physiological response to insulin stimulation of targeted cells, primarily these in the liver, muscles, and various types of adipose tissue. Insulin resistance impairs glucose release, leading to a compensatory increase in β-cell insulin secretion that, in turn, leads to a hyperinsulinemia state within the whole body. The metabolic impact of IR includes the resulting hyperglycemia, elevated blood pressure, dyslipidemia, visceral adiposity, innate and chronic inflammatory responses, endothelial layer function impairment, and dysregulation of the hemostasis balance [4,5,6]. Insulin resistance is believed to originate from muscle tissue with adaptive immune-related inflammatory states and a disbalance in free fatty acid metabolism, with subsequent ectopic lipid deposition [7]. An overview of the insights into the pathophysiology and outcomes of IR are shown in Figure 1.

Lifestyle intervention represents the first line of prevention as well as treatment for IR in prediabetic and diabetic conditions. Dietary intervention should be composed of a wide spectrum of calorie restriction patterns and a reduction in high-glycemic-index carbohydrates [8,9]. Physical activity has also been proven to improve both calorie consumption and insulin sensitivity in various tissues [10]. Many potential intervention targets and combinations with therapeutic activity have been described in recent years. A proof of principle for a non-peptide insulin-mimetic has been shown by specific activation of the intracellular B-subunit of the insulin receptor. An increment of insulin activeness has been achieved with molecules through enhancing phosphorylation and perpetuating the crucial kinases activity of the insulin receptor, as well as its protein substrates, after stimulation by insulin. Additional approaches involve promoting the activity of phosphatidylinositol 3-kinase and other downstream elements and contributors of the insulin-signaling and metabolic pathways. Experimental modifications aiming to attenuate signaling-dependent irregularities affected by proinflammatory cytokines, certain adipocyte hormones, excess fatty acids, glucotoxicity, and negative feedback loops by multifaced signaling chains have also proved the existence of feasible therapies. Numerous hormones, key metabolic enzymes, vitamins and minerals, co-factors, and transcription co-activators have shown insulin-sensitizing potential [11]. Despite the undebatable progress in this area, the treatment of IR is still challenging for scientists, medical doctors and all the people affected, directly or circumstantially, by this disorder. The present review paper highlights the most recent findings in the field.

## 2. Materials and Methods

**Figure 2:**
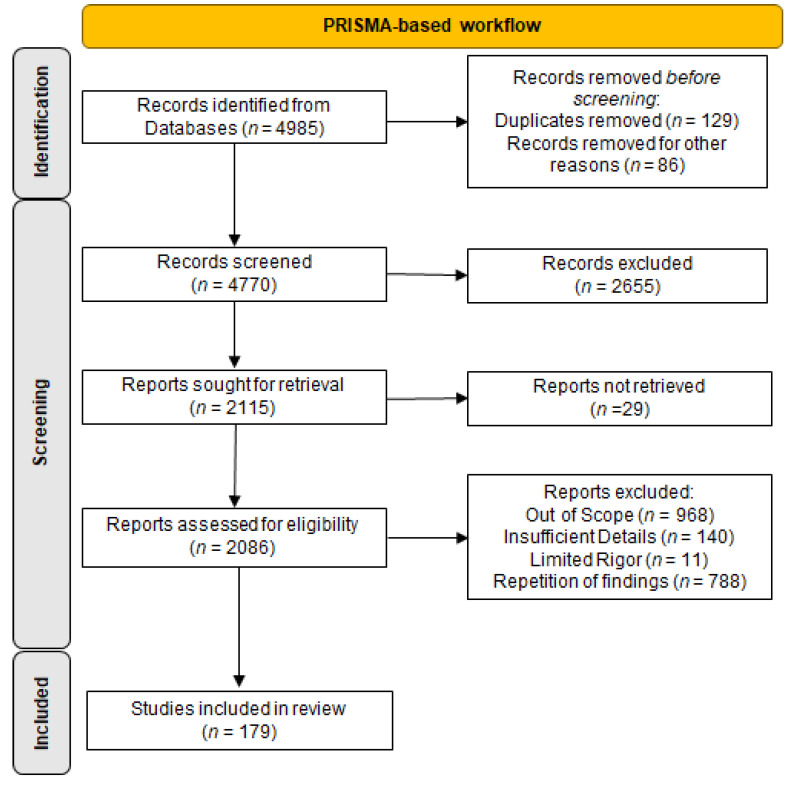
PRISMA-based scheme describing the search strategy.

A literature search was carried out using the MEDLINE/PubMed, Google Scholar, and SCOPUS databases, with a combination of keywords and MeSH terms, and papers published from February 2021 to March 2022 were selected and considered as recent advances in the area (*n* = 123). Moreover, a clinical trials registry (ClinicalTrials.gov accessed on 7 March 2022) was searched for clinical studies relevant for the paper and meeting eligibility criteria as described below. For providing insightful scientific background for the paper and for a comprehensive discussion, older papers were included as well and weighed as supportive papers (*n* = 46). The main inclusion criteria were publishing timeframe, focus on the treatment of IR per se, and high rigor of the research performed. Both basic and clinical studies were included. The search resulted in 4985 potential articles and 339 potential clinical trial registry outputs, as shown in Figure 2. After selection according to the eligibility criteria, availability of full texts, non-overlap with studies already included, and availability in English, 169 studies and 10 clinical trials fulfilled the criteria, were included and are discussed in the present paper. The literature research was carried out according to PRISMA guidelines [12] for narrative review, and a detailed chart of identification, screening, and inclusion is provided as Figure 2. To minimize the risk of bias, every potential paper was independently reviewed by two Authors [M.W. and S.P.], and in case of differing views, the lead author [T.W.K.] decided. In cases of the inclusion of a paper that might introduce bias due to a relatively small number of participants, studies at a single center, using a not well-established method or protocol, or providing data contradicting previously published papers, this information was highlighted to provide readers with full awareness of these limitations. Another limiting factor that is commonly present in scientific publishing is the length of availability of the final version of a paper. To overcome this, we used three different sources for the papers.

## 3. Discussion

### 3.1. Gut Microbiome Revitalization Promotes IR Alleviation

The gut microbiome plays an important role in human health–disease status and influences the development of chronic diseases ranging from metabolic disease to gastrointestinal disorders and cancers [13,14]. Recently published papers underlined the crosstalk between the microbiome and metabolic disorders leading to IR and related conditions. Hence, the modification and enrichment of the microbiome has gained attention as a promising target for proper insulin metabolism restoration [15,16,17,18]. Wu et al. provided some evidence that the administration of sesquiterpene glycosides isolated from loquat leaves leads to a decrease in the level of proinflammatory cytokines, as well as preventing IR occurrence via a modification of the gut microbiota in a db/db mouse model. They showed that seven key genera in the mice with sesquiterpene glycoside supplementation were enriched: *Lactobacillus*, *Lachnospiraceae_NK4A136_group*, *Ruminococcus, Bacteroides*, *Prevotellaceae_UCG-001*, *Alistipes*, and *Roseburia* [19]. A similar target for novel microbiota-related therapy was studied extensively by the group of Ma and colleagues [20], who investigated the potential beneficial action of a genetically modified version of the commonly known *Escherichia coli* Nissle 1917 (EcN-GM) in obese wild-type mice during an 8-week long supplementation [20,21]. The main outcomes of this study include, but are not limited to, an alleviation of hepatocyte steatosis and an increased level of glucagon-like peptide-1 (GLP-1) in serum, as well as decreased leptin and IR in these mice. The importance of gut microbiota in ameliorating IR has also been underlined by evidence that antibiotic treatment abrogated the protective effect of insoluble yeast β-glucan on high-fat diet (HFD)-induced obesity with accompanying IR [22,23]. A recombinant analog of leptin—metreleptin—is currently being studied during clinical trials (NCT00085982) to evaluate if 1 year of metreleptin usage will improve glycemia control in patients with genetic defects of insulin receptors, as well as to determine mechanisms by which metreleptin improves glycemia [24]. The importance of the gut microenvironment for maintaining physiological levels of glucose and insulin has been validated in the streptozotocin (STZ)-induced diabetes model in mice by adjuvant therapy with Sacha inchi (*Plukenetia volubilis* L.) tea [25]. Lin et al. showed that the six-week-long administration of an extract from Sacha inchi to mice with experimentally induced diabetes caused protective histopathological transformations in the pancreatic sections, leading to an attenuation of IR and improving insulin sensitivity in multiple tissues. The mechanism of this action seems to be related to the amelioration of gut microbial structural disorder and the enrichment of functional bacteria [25]. Interestingly, another team pointed out that Sacha inchi intake led to decreased proinflammatory states as well as oxidative stress augmentation and seemed to exert beneficial effects towards reducing the risk of metabolic disease [26]. Similarly, *Lactobacillus plantarum* SHY130 administration readjusted intestinal flora structure, enhanced the abundance of short-chain fatty acid (SCFA)-producing bacteria, such as *Faecalibaculum*, *Odoribacter*, and *Alistipes*, and increased the levels of SCFAs in diabetic mice, and it also ameliorated pathologically high glucose levels in HFD/STZ-induced diabetic mice by regulating the enteroinsular axis [27]. Furthermore, *Faecalibacterium prausnitzii*, a highly abundant butyrate-producing bacterium, has been proposed both as a biomarker for the development of different gut pathologies and as a potential treatment of insulin metabolism disturbances, due to its production of anti-inflammatory metabolites linked with Toll-like receptors and nuclear factor kappa B (NF-κβ) signaling [28,29]. A confirmation of the dependence of IR on NF-κβ has also been proven in a study evaluating the effect of sarsasapogenin in high-fat diet-fed mice, where an inactivation of the IKK/NF-κB and JNK inflammatory pathways led to ameliorated IR [30]. Progress in understanding the role of microbiota as a helper in the treatment of metabolic disorders has also been delivered by the team of Hua et al., who showed that a newly formed compound—P7C3-A20—has the ability to increase levels of NAD+ and alleviated NAFLD through promoting fibroblast growth factors (FGF) 1 and 21 in an LKB1/AMPK/CRTC2-conditional manner, leading to the growth and a re-balancing of gut microbiota [31]. Interestingly, FGF21, previously associated with overall metabolism, psoriasis, and embryonic development, has been proposed as an IR-alleviating factor by its downregulation during resistance training. This effect has also been linked to decreased levels of circulating myostatin and seems to be significantly beneficial in elderlies [32,33,34]. However, the findings evaluating the role of FGF21 in IR should be interpreted with care, since they seem to be more complex in terms of nutrition than expected [35].

A new insight into preventing the occurrence of IR in prediabetic conditions was presented by Ren and colleagues [36]. Utilizing next-generation sequencing and intestinal microbiota sequencing, they determined the effect of 1-Deoxynojirimycin (DNJ), the major alkaloid in *Morus alba* L., on the development of IR as well as cytokine profile changes in a murine model. Their work revealed that DNJ decreases the blood glucose level and improves insulin sensitivity, and thus might exert a preventive effect towards the progression of IR. Mechanistically, the observed effect seems to be, at least partially, dependent on suppressing the circulating levels of lipopolysaccharides (LPS), interleukin 6 (IL-6), and tumor necrosis factor α (TNF-α) in plasma, as well as suppressing the expression of suppressor of cytokine signaling 3 (SOCS3) and the activity of toll-like receptor 4 (TLR4)/NF-κB signaling pathways. Interestingly, a decreased abundance of *Enterococcaceae* and *Lachnospiraceae* was observed, suggesting their importance in IR [37]. At the same time, Ferreira et al. [38] proposed a similar strategy for preventing the dysregulation of insulin secretion and its tissue sensitivity in rodents. The administration of a fruit-derived probiotic formulation of *Limosilactobacillus fermentum* strains aiming to improve gut microbiota led to the mitigation of hyperlipidemia, insulin resistance, and sympathetic hyperactivity in rats fed an HFD. Since the exact mechanism of action remains unknown, the level of relevance is debatable, but this strategy seems to be a valuable option for treatment in subjects with cardiometabolic disorders. The above data show that our gut microbiota might be considered as a true ally in the battle with the progression and the occurrence of IR [39,40]. An interesting approach, based on the restoration of microbiota by 8-week long oral supplementation of glutamine—one of the key amino acids—is currently evaluated in a clinical study, and the investigators hypothesize that glutamine intake may contribute to improving glycemic control and IR-related symptoms [41].

### 3.2. New Mechanistic Insights into IR Treatment and Prevention

**Figure 3:**
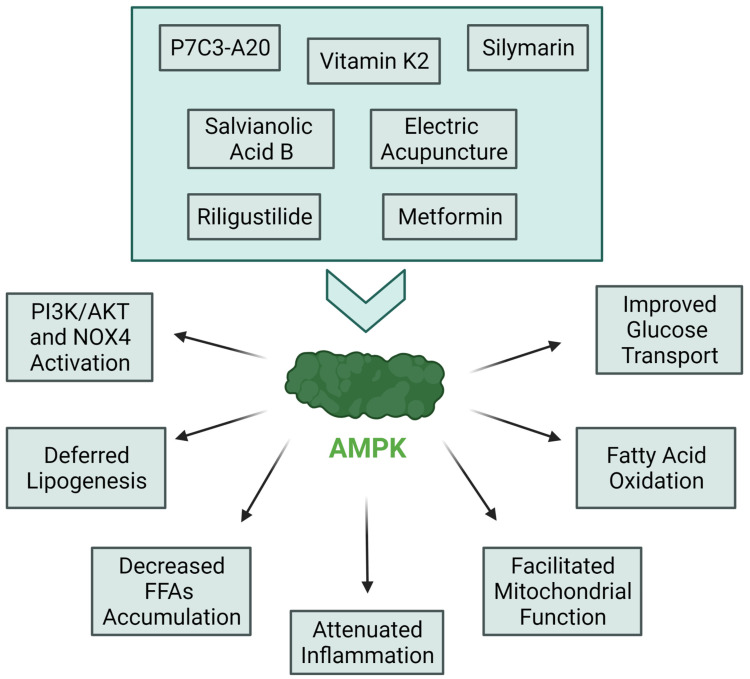
Schematic graph showing the effects of various agents on AMPK signalling with their subsequent impacts. Recently, many different agents targeting the AMPK-related pathways have been proposed as a novel approach for IR treatment. Using them leads to improved glucose and lipid metabolism and attenuates inflammation and oxidative stress parameters, as well as stimulating molecular pathways that are protective against IR progression [52,53,54,55,56,57,58,59].

Thought-provoking mechanistic insights into IR complexity have been proposed by Lyu et al. [42], who showed that the phosphorylation of an sn-1,2-diacylglycerol/epsilon isoform of protein kinase C (PKCε)/insulin receptor at threonine at amino acid position 1160 promotes white adipose tissue IR in a mouse model. This finding is relevant for short-term IR induced by an HFD, as results showed that insulin retained the ability to suppress white adipose tissue lipolysis after a 7-day HFD. Lule et al. [43] revealed new hypothetical mechanisms for a protective role of Exenatide, a widely used GLP-1 receptor agonist, for the amelioration of IR-induced inflammation and non-alcoholic fatty liver disease (NAFLD). Namely, their study pointed out a significant effect in decreasing the degree of steatosis, decreasing the SOCS3 expression level, and increasing the Irs-1 expression level in Wistar rats treated with Exenatide. An interesting update for the action of metformin on IR was delivered by Khodadadi and Dludla [44,45,46]. Since the exact action mechanism behind the glucose-lowering effect of metformin is not clear, the authors proposed that metformin exerts its effects through the inhibition of mitochondrial respiratory chain complex 1 and the activation of AMP-activated protein kinase (AMPK). AMPK has been recognized in the past as a potential IR-related pathway [47], and novel findings confirm this as shown on Figure 3 [48]. It is worth noting that the mechanism mentioned is one amongst many exerted by metformin to prevent IR in humans. In support of this, the confirmation of a mitochondrial-dependent approach for IR treatment is broadly discussed by Yang and colleagues, who state that mitochondrial membranes are critical to regulating glucose-related metabolic processes [49]. Protective effects of metformin might be intensified by combining metformin with silymarin. Hüttl et al. [50] showed that the additive effect of silymarin in metformin therapy can mitigate fatty liver development in pre-diabetic states and before the onset of IR, due to positively affected transcription factors involved in lipogenesis (*Scd-1*, *Srebp1*, *Pparγ*, and *Nr1h*) and fatty acid oxidation (*Pparα*) [51]. Simultaneously, the significance of AMPK signaling was confirmed by Entezari et al. [52], who demonstrated the role of AMPK-signaling in enhancing insulin sensitivity and in inhibiting oxidative stress as well as cell death in β cells. Moreover, the authors underlined the importance of the AMPK pathway crosstalk with crucial molecular pathways, namely: PI3K/Akt, NOX4, and NF-κB. These findings are also supported by papers published in the past that proposed an involvement of AMPK signaling in insulin biology, speculating that these mechanisms had IR-protective consequences [53,54,55]. AMPK pathway targeting [56] was also investigated by Liu et al. using an alternative medicine approach, namely, electric acupuncture, which was proposed as an adjuvant therapy for hepatic IR targeting of AMPK via the rapamycin complex 1 (mTORC1)/ribosomal protein S6 kinase, 70-kDa (p70S6K) signaling pathway in a Zucker Diabetic Fatty rat model. Results show that despite the debatable role of acupuncture, it might be considered an effective way to prevent IR and improve energy metabolism per se. Another noteworthy approach to protect against hepatic IR seems to be the total knockdown or pharmacological inhibition of chromosome 9 open reading frame 72 (C9orf72), which that has been found [57] to be an inhibitor of lipophagy by activating the Cdc42/N-WASP axis to facilitate hepatic IR. An additional approach for hepatic IR treatment is riligustilide, which upregulates the AMPK-TORC2-FoxO1 axis to attenuate gluconeogenesis in vivo and in vitro, thus preventing hyperinsulinemia and IR [58,59].

Similar results, showing the importance of down-regulating apoptosis and mitophagy pathways in preventing the occurrence of obesity, hyperglycemia, and IR in db/db mice were shown by Xiang and colleagues [60]. The protective role of salvianolic acid B in the progression of IR, diabetes-induced vascular endothelial dysfunction, and in the amelioration of hyperlipidemia, hyperglycemia, and hyperinsulinemia has been shown. Angiopoietin-like 3 (ANGPLT3) has emerged as an important regulator of lipid and glucose metabolism as well as insulin sensitivity [61,62,63]. Foss-Freitas et al. assess the efficacy and safety of targeting ANGPTL3 with vupanorsen in patients with familial partial lipodystrophy. They show a 55% reduction in the adipose tissue IR index, while other insulin sensitivity indices and glycated hemoglobin (HbA1c) levels were not changed [64].

### 3.3. Most Notable Innovations in IR Treatment Targeting Molecular Pathways

Spexin is a novel neuropeptide playing an emerging role in metabolic diseases [65,66]. Recent evidence introduces Spexin as a potential drug target for the development of new pharmacological strategies to cure hyperglycemia and IR clinically [67,68]. The proposed mechanism of action depends on abolishing the elevation of glucose consumption-related genes by the saccharomyces cerevisiae (GAL2) receptor antagonist or the silencing of the GAL2 receptor in myotubes. It reveals a new role for the GAL2/glucose transporter type 4 (GLUT4) signal pathway in protection against IR and an increase in glucose consumption in skeletal muscles with Spexin [69]. The role of GLUT4, as a factor involved in preventing and alleviating IR patterns, mainly due to IRS/Akt/GLUT4 pathway restoration, has been proven by Prasatthong et al. [70]. Not only has GLUT4 gained attention recently, but GLUT2 has also been shown to be the mechanism of action for using berberine [71]. This particle derived from the Coptidis rhizome has been reported to prevent abnormal glucose levels by shifting the location of PLC-β2 to the membrane, leading to decreased GLUT2 translocation and a normalization of glucose metabolism. Another noteworthy mechanism delaying the developmental programming of obesity and insulin metabolism impairment in the offspring of obese mothers is the inhibition of dipeptidyl peptidase IV (DPPIV), a ubiquitous enzyme that acts on incretin hormones, mainly GLP-1 and gastric inhibitory peptide, which maintain glucose homeostasis by increasing insulin secretion and decreasing glucagon secretion [72]. Montaniel et al. found that the activation of DPPIV preceded the progression of obesity, glucose intolerance, and IR in the male offspring of HFD-fed mothers [73]. The usage of its inhibitor, sitagliptin, delayed the progression of obesity and insulin metabolism abnormalities in male offspring. Interestingly, sitagliptin had no effects on females, thus, a maternal inhibition of DPPIV has the potential for addressing the transgenerational effects of maternal obesity [74]. Kim and colleagues studied the effects of vimentin deficiency, providing us with the interesting finding that it prevents obesity and insulin resistance in mice fed an HFD. Moreover, they come to the very bold conclusion that vimentin is central to linking obesity with diabetes—this statement needs to be validated carefully [75]. A decidedly new approach to managing glucose tolerance and reducing the risk for IR onset was proposed by Okano and colleagues. They adapted the STZ-induced diabetes mouse model to study the effect of thrombomodulin on glucose metabolism and insulin secretion. Thrombomodulin, which is mainly known as an intrinsic regulator of the coagulation cascade, significantly improved glucose tolerance, increased insulin secretion, and decreased pancreatic islet areas of apoptotic β-cells. Moreover, an eight-week-long treatment with humanized thrombomodulin enhanced the proportion of regulatory T cells and tolerogenic dendritic cells in the spleen compared to counterpart diseased mice treated with saline [76,77]. Recently published articles have shown the existence of a very tight connection between metabolic disorders, with particular emphasis on IR, and hemostasis disturbances [78,79]. A balancing of the key metabolic processes like glucose control, lipid-lowering, and weight loss also normalizes coagulation disorders in diabetes patients. Intriguingly, glucose-lowering drugs, especially those with proven cardiovascular beneficial agents such as GLP-1 receptor agonists and sodium-glucose co-transporter inhibitors, have been shown to exert direct anticoagulation effects in patients with diabetes, thus allowing new strategies in the treatment of diabetic comorbidities [80].

Very interesting mechanical insights into insulin metabolism regulation were brought forward by a team from Lund University who evaluated the role of the overexpression of β-cell cocaine- and amphetamine-regulated transcript (CART) in insulin secretion and glucotoxicity [81]. Using in vitro cell cultures and amurine model, they showed that β-cell CART acts to stimulate insulin secretion when the β-cell function is challenged. They proposed that the increase in β-cell CART is part of compensatory mechanisms trying to counteract hyperglycemia in the course of T2DM. This is an interesting finding since the role of CART-based agents as a therapeutic modality in type 2 diabetes has previously been proposed [82,83]. Another mechanistic approach to preventing and treating IR has recently been proposed by Yazdanimoghaddam et al. [84], aiming to normalize the levels of magnesium that are significantly lower in the course of T2DM. Using a rodent model, the authors showed that supplementation of the daily diet with magnesium sulfate led to a decrease in triglycerides as well as oxidized-LDL, and improved serum insulin and irisin levels. As stated by the authors, the lowering of the oxidized-LDL fraction seems to be the key player in decreasing the prevalence of IR. Magnesium has been shown to be a contributor in alleviating metabolic syndrome, IR, and excessive body weight by Askari [85]. Another study evaluating the role of a macro element, namely calcium, showed that calcium-fortified beverages have a beneficial effect on insulin sensitivity and some antioxidant enzymes in healthy elderly people [86]. Not only minerals are in the focus of researchers. Vitamin K2 also gained attention, by showing its capability to decrease IR in the skeletal muscles on the sirtuins-dependent pathway. The exact mechanisms combine the role of the mitochondrial AMPK pathway as well as restoring their function based on transcellular sirtuins signaling [87]. Furthermore, vitamin D_3_ has also been proposed as a promising adjuvant for metabolic syndrome treatment. In a rodent model of induced dyslipidemia, hyperinsulinemia, IR, and impaired glucose tolerance, supplementation of this vitamin led to a significant reversal in the observed pathological conditions, suggesting vitamin D_3_ as an auspicious therapeutic target for glucose metabolism disbalance [88].

A distinct, innovative approach to cracking the enigma of IR has been proposed by Manickam et al. [89] in studying nicotinamide phosphoribosyl transferase (Nampt), a key enzyme in the NAD salvage pathway, the levels of which are decreased in various metabolic diseases [90]. Using Nampt^+/−^ mice and a specific Nampt activator—pool 7, compound 3 (P7C3)—the authors provided a piece of evidence that P7C3, through activating Nampt, improves type 2 diabetes and that gastrocnemius muscles display a significant decrease in inflammatory lipid mediators. Congruent findings were presented by Khan et al. [91], who showed the involvement of endoplasmic reticulum-originated oxidative stress in the progression of hyperglycemia and IR in renal glycemic toxicity. An in-depth analysis of the mechanistic background revealed that the toxic effect of overloaded glucose capacity is mediated by the Tribbles 3 (TRB3)-Forkhead box O1 (FoxO1) signaling pathway via upregulation of initiation Factor 2α (p-eIF2α), X Box Binding Protein 1 spliced (XBP1s), Activating Transcription Factor 4 (ATF4) and C/EBP Homologous Protein (CHOP) expression [91]. Another alluring mechanistic insight into IR biology was provided by Patti and his team, who pointed out another mechanistic insight into IR biology—the receptor for free fatty acid, which is widely known for its regulating action in metabolic and anti-inflammatory processes through their action on incretins [92]. In their study, the evidence for a protective role of the receptor agonists was proven. Namely, the activation of receptors increases insulin sensitivity, induces body mass loss, reduces inflammation, and has beneficial metabolic effects. Stimulating data has been shown in a paper by Fehsel et al., who decided to investigate the pro-IR side effect of clozapine, which has been associated with aryl hydrocarbon receptor (AhR) activity. AhR was considered in the past as an endogenous receptor mediating the effects of dioxin. After years of studies, it has been shown that AhR plays a role in mediating many different signals due to its cross-talk with p38-MAPK, VEGF, Mas-Receptor, and nicotinamide adenine dinucleotide phosphate (NADPH) [93]. The study has shown that clozapine triggered AhR activation, thereby impairing adipogenesis and vasorelaxation in HepG2 cells and adipocytes, which in turn favors the progression of obesity and IR-related metabolic disturbances [94]. An interesting target for preventing IR and T2DM seems to be phosphatidylinositol 4-phosphate 5-kinase type I c, which is known for regulating focal adhesion formation, invasion, and cell migration signal transduction cascades with the crosstalk with intercellular adhesion molecule-1 (ICAM-1) and mTORC1-induced upregulation of the glycolytic pathway, and also favors the differentiation of specific inflammatory Th cell subsets [95,96]. Using adipose-specific conditional knockout mice, it has been proven that this knockout significantly reduced IR burden as well as hyperlipidemia. Confirming this, in vitro studies showed that adipogenesis was markedly impaired in the mouse stromal vascular fraction (SVF) from phosphatidylinositol-4-phosphate 5-kinase type 1 gamma (PIP5K1c)-deleted mice [95]. Another novel target to prevent IR involving the down-regulation of the Lysine Acetyltransferase 7 (*KAT7*) gene was recently proposed by Wang et al. [97]. Their results proved that knockdown or inhibiting *KAT7* led to significantly increased insulin sensitivity in in vivo and in vitro models. Interestingly, the knockdown of *KAT7* seems to reduce inflammation and oxidative stress caused by aging. These findings are amongst others indicating that KAT7 might be used as one potential target for the treatment of IR caused by aging [97]. Another gene-based approach has been proposed by Haas et al., who evaluated the role of apolipoprotein A-I (*apo A-I*) gene stimulation by sugars on insulin metabolism in hepatic and intestinal derived cell lines [98]. They concluded that D-allulose and D-tagatose—commonly used sweeteners—may have favorable effects on IR and obesity progression. As a proposed mechanism of action, they showed that D-allulose and D-tagatose maximize *apo A-I* production via increased transcription factor specificity protein 1 (SP1)-binding to the insulin response element of the promoter. Additionally, these sugars regulate some insulin-responsive genes, increase the production of apo-A-I, and additionally might exert anti-atherogenic properties.

### 3.4. Well Known Molecules with Newly Discovered Anti-IR Features

The year 2021 brought also some preliminary evidence revealing previously unknown properties of well-known molecules towards the up-or-down regulation of mechanisms contributing to IR. Yaryari et al. [99] linked levels of asprosin, white adipose tissue-derived glucogenic adipokine, to the development of obesity, insulin resistance, and metabolic disturbances related to valproic acid treatment in epileptic patients. This is a very valuable observation since epilepsy and T2DM are interrelated and approximately 55% of epileptic patients are diagnosed with obesity [100]. Grapentine et al. were the first to determine that decreased levels of phosphate cytidylyltransferase 2 (Pcyt2), the rate-limiting enzyme in the synthesis of phosphatidylethanolamine, are correlated with the risk of IR [101,102]. Using Pcyt^2+/−^ mice they showed that supplementation with the substrate of Pcyt2, phosphoethanolamine, leads to improved fatty acid metabolism and insulin signaling, as well as attenuating inflammation, thereby reducing the risk for the occurrence of IR. Melatonin synchronizes central but also peripheral oscillators (pancreas, liver, kidney, fat, gut, etc.), thereby allowing the temporal organization of biological functions through circadian rhythms [103]. In their review paper [104], Wang and colleagues concluded that melatonin can protect from kidney damage caused by obesity and hyperglycemia by inhibiting inflammation and oxidative stress, revealing its therapeutic potential towards halting IR progression leading to kidney disfunction. Reticulon-4 (Nogo) is an endoplasmic reticulum-resident protein and the inhibitor of neurite outgrowth specific to the central nervous system [105]. The team of Dr. Yang [106] showed a novel role for Nogo using HFD-induced obese mice that were injected with scrambled or Nogo siRNA. The results indicated that a reduction in Nogo levels led to a protective effect against HFD-induced obesity, reversed whitening of brown adipose tissue, and an attenuation of inflammatory responses in organs due to enhanced NF-κB p65 degradation via the lysosome pathway. Clenbuterol [107] is commonly known for its β2-agonistic properties and approved in the United States for veterinary use in non-food animals. Nevertheless, clenbuterol has gained increasing attention due to its illegal usage by bodybuilders and fitness enthusiasts attracted to its hypertrophic and lipolytic effects [108]. Considering that systemic treatment with β2-adrenergic receptor agonists results in multiple beneficial metabolic effects, including improved glucose homeostasis, Meister et al. [107] hypothesized that it might be used as an agent to prevent IR and regulate glucose metabolism. The preliminary results show that clenbuterol administration caused pronounced improvements in glucose homeostasis and prevented metabolic deficits in mouse models of β-cell dysfunction and IR via activation of skeletal muscle β2-adrenergic receptors and the stimulatory G protein called Gs [107]. Jeddi et al. revised the role of nitric oxide in type 1 diabetes mellitus (T1DM)-induced osteoporosis proposing the role of nitric oxide donors as an alternative strategy to halt not only osteoporosis during T1DM, but also IR per se [109]. Baicalin, along with its aglycone baicalein, is a positive allosteric modulator of the benzodiazepine site and a non-benzodiazepine site of the γ-aminobutyric acid type a (GABA_A_) receptor [110]. This compound has been found to protect against IR and metabolic dysfunction mainly through activation of the GALR2-GLUT4 signal pathway [111]. Interesting insights into IR prevention were brought forward by Ghoshal et al. [112] by proposing targeting the cytochrome P450 epoxygenase Cyp2c44, a major epoxyeicosatrienoic acid (EET)-producing enzyme, as a form of prevention of IR. In their study, the water-soluble EET analog restores insulin signaling in vivo in Cyp2c44(−/−), thereby enhancing hepatic insulin signaling, a decreased expression of gluconeogenic genes, and an increased expression of glycogenic genes. Mechanistically, they show that insulin-stimulated phosphorylation of insulin receptor β (IRβ) is impaired in primary Cyp2c44(−/−) hepatocytes and that this can be restored by cotreatment with EET-A and insulin, leading to diminished IR and risk of IR progression [112]. The kynurenine pathway, a primary downstream catabolism route for dietary tryptophan has been described as a potential target for IR prevention [102,113]. The proposed interaction takes place due to kynurenine pathway activity leading to increased and persistent inflammatory state generation that facilitates IR progression.

### 3.5. Revision of New Strategies in IR Prevention Using Nature-Derived Compounds

Diet enrichment with marine oil from *Calanus finmarchicus* has been shown as an alternative strategy for overcoming the burden of IR in prediabetic conditions in a single-center patients’ study. After a 12-week long intake of Calanus oil, a significant improvement in hepatic IR index, homeostatic model assessment for insulin resistance (HOMA-IR), and fasting insulin levels were observed. However, the mechanisms as well as the active compound formulation are only speculative at this stage [114]. Furthermore, flaxseed oil, a source of antioxidants and anti-inflammatory compounds, used along with hesperidin was shown to improve metabolic syndrome outcomes and normalize glucose metabolism [115]. One more piece of evidence showing nature-based medication as a possible treatment for IR is the saponin-rich extract from Tribulus Terrestris [116]. The previously known anti-inflammatory action of the multi-compound extract was adopted to be used in an HFD-fed rat model to assess its impact on glucose metabolism and chronic inflammatory states. The results showed that the anti-inflammatory properties of saponins from Tribulus terrestris helped in the alleviation of IR and body weight gain in obese female rats. Another molecule targeting oxidative disbalance and alleviating mild inflammation states, thus preventing the occurrence of IR in a rats model, is auxin [117]. Similar results have been obtained using puerarin [118]—the isoflavone that is widely used in China for the treatment of cardiovascular and neurodegenerative diseases. Colchicine, isolated from the autumn crocus (*Colchicum autumnale*), has been proposed as an anti-IR acting agent and clinical studies started in November 2021 to study whether colchicine improves metabolism amongst patients with high body weight, increased inflammation, and high insulin in the blood, but who have not yet developed high blood sugar [119]. Noh et al. [118] identified the puerarin treatment-targeted genes and molecular docking with puerarin: TNF-α, M1, and M2 macrophages based on functionally enriched pathways. In their murine model, they showed that puerarin significantly improved fat pad weight, adipocyte size, fat area in the liver, free fatty acids, triglycerides, total cholesterol, and HDL-cholesterol in vivo. Another plant-derived substance with a high potential to counteract IR is indicaxanthin—a type of betaxanthin with high anti-oxidative capabilities—isolated from the fruit of *Opuntia ficus-indica* [120]. Indicaxanthin induced significant, beneficial effects on HFD-induced glucose dysmetabolism, reducing fasting glycemia and improving glucose and insulin tolerance as well as recovering the HOMA index to physiological values. Effects observed in this study seem to be dependent on the attenuation of TNF-α, c-c motif chemokine ligand 2 (CCL-2), and F4-80 gene expression, as well as restoring the oxidative balance by harmonizing iNOS and COX-2 levels [121]. Further, oleanolic acid, a triterpene that is abundantly present in olive leaves, has been studied in a clinical trial as a possible nature-based agent in the prevention of metabolic syndrome due to its anti-inflammatory activity [122]. Another notable way to counteract IR seems to be supplementation of polysaccharides from *Angelica sinensis,* which have been shown to preclude hepatic IR through the inhibition of the phosphorylation of c-jun n-terminal kinase (JNK) and P38 produced by the receptor for advanced glycation end products (RAGE) small interfering RNA (siRNA) in IR-sensitive hepatic G2 cells [123]. To keep the review comprehensive, we summarise that several recent studies show different natural compounds with beneficial effects towards counteracting IR and normalizing glucose and insulin metabolism, namely: pomegranate peel anthocyanins [124], *angelica acutiloba* extract [125], a traditional Chinese medicine formulation called *Yiqihuoxue* [126], Ginsenoside Rb1 [127], Cassia seed extract [128], and Lanzhang Granules [129].

### 3.6. Dietary Habits and Physical Activity Play a Crucial Role in IR Treatment

The importance of maintaining good eating habits to prevent or diminish the burden of IR is not debatable [130]. A recently completed clinical study, CALERIE™, showed that a reduction in daily calorie intake improves lipid-related emerging cardiometabolic risk factors in healthy adults without obesity [131] by reducing inflammation and branched chain amino acids and shifting lipoproteins from atherogenic to cholesterol-transporting. Another relevant study published by Allehdan et al. [132] showed that carbohydrate-counting and carbohydrate-counting combined with the DASH diet led to improved fasting blood glucose and 1-h postprandial glucose levels in pregnant women with DM. It confirms that a well-balanced diet decreases the risk of IR occurrence as well as reducing DM-related complications [133]. Partially filling this gap, it has been prospectively documented that adherence to the MedDiet, including vegetables, fruits, oilseeds, extra virgin olive oil, and fish, combined with engagement in high levels of physical activity, had multiplicative effects on all-cause mortality risk reduction in a Spanish population (multivariable-adjusted hazard ratio = 0.66, 95% confidence interval: 0.46–0.96) [134,135], in which incident deaths were related to cancer and cardiovascular events, two conditions frequently found in patients with IR and T2DM [136]. Intermittent fasting significantly reverses diabetes, thyroid, elevated blood pressure, and high blood lipid levels, and leads to the normalization of the body mass index; studies have also shown that it has been followed or instructed for the treatment and prevention of cancer and neurodegenerative diseases with dietary interventions [137]. Further evidence underlining the role of proper diet is provided by a meta-analysis showing that enough intake of vitamin D has shown beneficial effects on fasting blood glucose, HbA1c levels, and fasting insulin evaluation in prediabetics [138,139]. Charatcharoenwitthaya et al. showed that a low protein intake increases the odds for insulin resistance. Contrarily, a high intake of full-fat dairy products and dietary fiber has been associated with a potential protective effect against IR [140]. These findings led researchers to start clinical trials to investigate whether slowly fermentable fibers are expected to have a superior potential for influencing host-microbiota by improving adipose tissue function, normalizing lipid metabolism, and hepatic fat accumulation and thereby improving insulin sensitivity [141]. An interesting aspect regarding the care of high-risk patients has been raised by Zou et al. [142] in showing that high-quality nursing can effectively improve the blood glucose levels and psychological states of patients with DM and contributes to higher treatment compliance. Surprising results from a study on Chinese adults showed that [143] high-frequent tea consumption increased the risk of metabolic syndrome among older Chinese adults. These findings may add novel knowledge to current studies regarding the controversial effect of tea consumption on cardiovascular and metabolic health among the aged population. Furthermore, Lyu et al. demonstrate the importance of the PM sn-1,2-DAG/PKCε/Insr Thr1160 phosphorylation pathway in mediating lipid-induced WAT IR and represent a potential therapeutic target to improve WAT insulin sensitivity [42,144]. Attractive findings showed that an overload of amino acids in daily food intake leads to excessive activation of mTOR, leading to desensitization of insulin receptors and thus reducing insulin-mediated glucose uptake [128]. Sheng et al. also showed that restrictions in calorie intake attenuate age-related metabolic disorders and proposed a calorie-low diet as an effective approach to metabolic health during aging [145]. Further noteworthy findings were provided in a paper by Escalante-Araiza, who showed that Mexican food, rich in antioxidants, vitamins, and minerals, might improve the metabolic status in populations that are at risk of developing metabolic syndrome [146]. In 2024, a clinical trial (NCT02193295) is expected to finish up and provide evidence on whether a small weight loss in lean, insulin-resistant offspring of type 2 diabetic patients will improve insulin resistance [147]. A clinical trial focusing on a more intense approach is a study evaluating whether the body response to three weeks of intermittent fasting (alternate-day fasting) will result in a metabolic shift towards the use of ketone bodies compared to three weeks of a Western diet [148]. The investigators of both trials aim to introduce a new paradigm for prevention and attenuate the burden of IR. Similarly to eating habits, physical activity plays a major role as a prevention or alleviation factor for the occurrence of IR in the diabetic milieu. An in-depth review paper has aggregated the current state of knowledge and proven that strategies to integrate everyday physical activity into lifestyle treatment schedules might be a promising approach for improving the clinical outcomes of patients suffering from IR [149,150]. Worth mentioning is a currently ongoing clinical trial that aims to investigate the hypothetical clinical benefits of a newly designed circuit training based on strength and persistence exercises for the normalization of insulin sensitivity and vascular endothelial function [151]. Moreover, regular and well-balanced training might impact positively on the control of glucose metabolism also on the molecular level [152].

### 3.7. Cardiovascular Disease Treatment Alleviates IR Severity

Cardiovascular diseases (CVD) have been proven to interconnect broadly with the burden of IR [153]. Interestingly, many types of CVD treatment options also target molecules and pathways that are highly involved in the occurrence and progression of IR. The literature widely suggests that proper therapy aiming to improve CVD outcomes also normalizes lipid profiles, attenuates chronic inflammation and redox disbalance, controls insulin response in cells and leads to a decrease in overall mortality in various populations [154,155].

A recently published paper in *The Lancet* evaluating the role of anti-CVD pharmacological interventions in preventing T2DM and thereby IR showed that angiotensin-converting enzyme inhibitors and angiotensin II receptor blockers have the most favorable outcomes for these groups of patients [156]. Polyphenols that are broadly known for their anti-inflammatory and anti-oxidative action are commonly used as preventive agents for the occurrence of hypertension as well as improving cerebral blood flow. Recently, it has been proposed that polyphenols attenuate IR severity, along with slowing down its development [157]. These compounds decrease the activity of the IRS-1/PI3-k/Akt signaling pathway to reduce β-cells loss in the pancreas [158]. Moreover, polyphenols impact ATP-sensitive Ca^2+^ and K^+^ ions channels leading to an increase in insulin release. Stabilizing these channels also has a vital role in preventing CVD events related to arrhythmias and hypertension. Another recent study has shown that an anti-hypertensive agent, moxonidine, exerts additional beneficial metabolic changes in patients suffering from hypertension [159]. It has been shown that moxonidine normalized neuropeptide Y levels in serum and decreased hyperlipidemia, as well as decreasing the obesity-related risk of mortality and morbidity due to CVD. Similar evidence was provided by Kozono et al., who showed that antihypertensive therapy improves glucose metabolism and imbalances in proinflammatory cytokine expression in rats with hypertension, proposing that antihypertensive therapy acting through immunological factors may be beneficial for patients with impaired glucose metabolism and IR [160]. Other anti-hypertensive agents, namely losartan and levamlodipine besylate, have also been proposed as agents exerting beneficial effects for patients suffering from IR with diagnosed hypertension [161]. Contradictory results of using β-blockers in hypertensive patients at the risk of forming IR were published by Rane et al., who speculate that β-blockers decrease insulin sensitivity in hypertensive patients, leading to an increased prevalence of IR [162]. IR has recently been proposed as a marker/predictor of stroke risk in both diabetic and non-diabetic cohorts (95% CI: 69.77, 73.38 and 70.14, 73.79) [163,164]. Patients with ischemic stroke or transient ischemic attack (TIA) are usually treated with pioglitazone to reduce the risk of following strokes or myocardial infarctions [165]. A clinical trial has shown that usage of pioglitazone not only reduces the risk of CVD events, but also leads to improved insulin sensitivity as well as reducing the T2DM risk in general [165]. Interestingly, this drug is currently under clinical trial (NCT00015626) as a potential agent for an aggressive form of IR treatment [166]. Similarly, the usage of acetylsalicylic acid (ASA, aspirin) in the low dose-pattern to prevent prothrombotic events in various patients’ populations might have beneficial effects on glycemic control, reflected in lower levels of glycosylated hemoglobin and a reduced risk of IR development [167]. It has been shown that the use of ASA and Tofacitinib could mitigate insulin resistance and hyperglycemia in T2DM [168]. In recently published data including 237 patients with recent ischemic stroke or TIA receiving aspirin and/or clopidogrel, insulin resistance assessed by HOMA-IR was found to be related to high residual on-treatment platelet reactivity, which was not specifically restricted by clopidogrel resistance [169,170]. Although these results are only speculative at this stage, they shed a light onto the possibility of the involvement of antiplatelets agents in the modulation of IR.

One of the most important approaches for preventing CVD is physical activity. Each of the training modalities, namely aerobic and resistance exercise as well as leisure-time physical activity, have been shown to prevent the development of the major CVD risk factors [171]. The same findings are also accurate for IR prevention; thus, CVD patients that are at a higher risk of IR and follow exercise-based guidelines benefit dually. Similarly, the aforementioned DASH diet, which is considered as a gold standard for diabetic patients, is also recommended as a dietary pattern for decreasing the risk of CVD occurrence as well as improving outcomes during hypertension, stroke and vascular diseases [172,173].

### 3.8. Negative Results in Current Advances of IR Treatment and Prevention

To make this review comprehensive, there is also a need to mention some recent works that provided negative results. One of them is a paper by Ogawa et al. [174] aiming to evaluate the effect of electrolyzed hydrogen-rich water on oxidative stress suppression and the attenuation of IR in a multicenter prospective double-blind randomized control trial. Despite the existence of very solid evidence linking oxidative disbalance to IR [175], there were no significant differences in changes in IR, evaluated using the homeostasis model assessment of insulin resistance, between the study group and controls who drank filtered water as a placebo. Interestingly, another research paper showed that the estimated glucose disposal rate (eGDR) predicts all-cause mortality in type 2 diabetes, however, it does not directly link chronic kidney disease with IR. On the other hand, the authors speculate that the impact of IR in individuals with albuminuric kidney disease may be mediated by its relationship with albuminuria [176]. A very promising clinical trial evaluating the effectiveness of ladarixin (an investigational, oral, small-molecule inhibitor) in the treatment of IR and improving insulin sensitivity in overweight patients was unexpectedly terminated and withdrawn without further generation of result [177]. Ngo Nkondjock et al., in their well-executed cross-sectional study, surprisingly showed that homeostasis model assessment was not a mediator on calcium levels in serum-related hypertension in a group of 425 participants of the National Health and Nutrition Examination Survey [178]. Chromium is a tracing element that is believed to significantly improve overall metabolism balance and decrease glucose intolerance and hyperinsulinemia. White and colleagues used an HFD-STZ-induced Wistar rats model to check whether chromium may improve glucose and lipid metabolism. In their solid and comprehensive study, they showed that Cr (III) had no significant effect on glucose and lipid metabolism in diabetic rats [179].

## 4. Conclusions

Insulin resistance plays a crucial role in morbidity and mortality amongst diabetic patients, and therefore, new therapeutic strategies are necessary to provide a growing number of patients with better care and more effective treatment. Since diabetes mellitus research is considered as one of the fastest-growing fields of medicine, many different directional studies are performed every year to provide answers on how to restore the harmony in insulin status in the course of diabetes and in the diabetic milieu. One of the most widely available and strongly recommended approaches for achieving this aim is a change of dietary habits as well as improving the profile of physical activity. Recent findings have confirmed and shed new light on the benefits of these patterns. Moreover, the restoration and modification of gut microbiota seems to be a novel and robust recommendation for preventing and attenuating the burden of IR. Interestingly, a wide spectrum of natural compounds has been proposed for the treatment of IR. Many of them have been used successfully at the basic science stage and they might be considered as molecules that have to enter the clinical trials phase of development. Furthermore, it has been proposed recently that drugs which are commonly used and available on the market might also be considered as potential treatment options for IR. Summing up, all the data presented show that in the field of IR treatment described here, significant progress has been observed within the last year.

## Data Availability

Not applicable.

## Abbreviations

AhR	aryl hydrocarbon receptor
AMPK	AMP-activated protein kinase
ANGPLT3	Angiopoietin-like 3
apo A-I	apolipoprotein A-I
ATF4	Activating Transcription Factor 4
C9orf72	pharmacological inhibition of chromosome 9 open reading frame 72
CART	cocaine-and amphetamine-regulated transcript
CCL-2	c-c motif chemokine ligand 2
CHOP	C/EBP Homologous Protein
DM	Diabetes Mellitus
DPPIV	dipeptidyl peptidase IV
EcN-GM	*Escherichia coli* Nissle 1917
EET	major epoxyeicosatrienoic acid
eGDR	estimated glucose disposal rate
FoxO1	Forkhead box O1
GABAA	γ-aminobutyric acid type a
GAL2	saccharomyces cerevisiae
GLP-1	glucagon-like peptide-1
GLUT4	glucose transporter type 4
HbA1c	glycated hemoglobin
HFD	high-fat diet
HOMA-IR	homeostatic model assessment for insulin resistance
ICAM-1	intercellular adhesion molecule-1
IR	insulin resistance
IRβ	insulin receptor β
JNK	c-jun n-terminal kinase
KAT7	Lysine Acetyltransferase 7
mTORC1	rapamycin complex 1
NADPH	nicotinamide adenine dinucleotide phosphate
NAFLD	non-alcoholic fatty liver disease
Nampt	nicotinamide phosphoribosyl transferase
NF-κβ	nuclear factor kappa B
Nogo	Reticulon-4
p70S6K	ribosomal protein S6 kinase
P7C3	pool 7, compound 3
Pcyt2	phosphate cytidylyltransferase 2
p-eIF2α	Factor 2α
PIP5K1c	phosphatidylinositol-4-phosphate 5-kinase type 1 gamma
PKCε	protein kinase C
Pparα	fatty acid oxidation
RAGE	receptor for advanced glycation end products
SCFA	short-chain fatty acids
siRNA	small interfering RNA
SP1	specificity protein 1
STZ	streptozotocin
SVF	stromal vascular fraction
T1DM	type 1 diabetes mellitus
T2DM	Type 2 Diabetes Mellitus
TRB3	Tribbles 3
XBP1s	X Box Binding Protein 1 spliced
